# Ascites—V

**Published:** 1909-07-10

**Authors:** 


### MEDICINE.

### ASCITES—V.

### THE DIFFERENTIAL DIAGNOSIS OF THE CAUSE OF ASCITES.

GENERAL CONSIDERATIONS.

If ascites is the only fluid accumulation in the case, or if there is swelling and œdema of the feet as well, but the ascites appeared first; or if the ascites is out of proportion to the degree of dropsy elsewhere, it is most probably due either to one of the affections of the peritoneum itself, a blood disease, or else to one of the causes of portal vein obstruction.

If it is associated with general anasarca, such as œdema of the legs, body, and face, and with effusion into the pleura or pericardium, the most likely cause is Bright's disease.

If swelling or œdema of the legs and lower part of the body were the first symptoms to be noticed, and ascites followed, then chronic valvular or muscular disease of the heart, chronic lung disease, or obstruction of the inferior vena cava above, or involving the hepatic veins, would be likely. It should be remembered, however, that there are exceptions to this general rule, for ascites does not attract notice from the patient as readily as does œdema of the ankles and legs; and in some cases of cirrhosis of the liver, œdema of the legs may be complained of some time before the definite onset of ascites.

If jaundice is associated with ascites, it points to some form of portal obstruction in the liver itself or in the portal fissure. If the jaundice is intense, there is probably a serious lesion either in the glands in the portal fissure in the common bile duct, or in the head of the pancreas, thence involving the bile duct.

If the liver is enlarged, the ascites will probably be due to one of the following conditions:—

(*a*) Secondary carcinoma; (*b*) cirrhosis of the liver; (*c*) peri-hepatitis; (*d*) syphilis of the liver; (*e*) passive congestion and nutmeg change from chronic heart or lung disease with failing cardiac compensation.

If ascites is associated with the presence of multiple intra-abdominal tumours, either tuberculous or malignant peritonitis is probable.

It will now be well to say a few brief words as to the differential diagnosis of each of the various causes of ascites.

I. DISEASES OF THE PERITONEUM.

(*a*) *Simple Acute Peritonitis.*—By simple acute peritonitis is meant an acute inflammation of the peritoneum, which is followed by exudation of serous non-purulent fluid, and which is apparently due to no perforation of a viscus, or to any other definite local primary focus of disease. It is analogous to acute pleurisy with effusion, but is much rarer. It usually occurs as a complication of Bright's disease.

The onset as a rule is sudden with acute abdominal pain and vomiting. The abdomen soon becomes distended, and its walls are tender and rigid. The signs of free fluid in the peritoneal cavity are present. The pulse is rapid, and the temperature more or less raised. It may be mistaken for acute peritonitis, due to perforation of a gastric or duodenal ulcer, and the like, and laparotomy may consequently be performed. Recovery without operation is possible, but, as a rule, its association with extensive Bright's disease indicates that the end is near.

The acute onset and the coexistence of Bright's disease, with its typical urinary changes, are the most important diagnostic points. It cannot be too much emphasised that urinary examination should on no account be omitted in all acute abdominal cases.

(*b*) *Simple Chronic Peritonitis.*—By simple chronic peritonitis is meant a chronic inflammation which is neiher tuberculous nor malignant. It may result from repeated tappings for ascites due to another cause. One sees cases of mitral disease, for example, in which ascites occurs amongst other effects of failing compensation. Paracentesis abdominis becomes necessary. It may be repeated. The heart may thereafter recover perfect compensation, and all the symptoms except ascites disappear. Unless one thinks carefully, one may still attribute this ascites to the mitral disease, when in reality it is no longer due to this, but to the chronic peritonitis resulting from the tapping of ascites, due in the first place to the heart disease. It may also be associated with chronic Bright's disease, alcoholism, or syphilis.

The abdomen is usually distended with fluid, which may be free in the peritoneal cavity and give rise to general abdominal distension, or, on the other hand, may be limited to certain parts by adhesions and cause an enlargement of the abdomen, which is not symmetrical.

If the mesentery is shortened and the intestines are matted together and bound down to the spine, there may be dulness all over the abdomen, or even dulness in front, with some resonance in the flanks, so that ascites from this cause is very liable to be mistaken for ovarian tumour.

In some cases a peritonitic rub may be felt or heard on auscultation over the upper part of the abdomen, and there may be some corresponding tenderness on palpation. The patient may complain of continual dull pain in the abdomen, and there may be occasional vomiting, constipation, or diarrhœa.

The presence of albumen and renal tube casts in the urine may be very helpful in the diagnosis, and in some cases there may be the symptoms and blood counts corresponding with the blood diseases enumerated above, amongst the possible causes of chronic peritonitis and ascites.

